# NCBI's Conserved Domain Database and Tools for Protein Domain Analysis

**DOI:** 10.1002/cpbi.90

**Published:** 2019-12-18

**Authors:** Mingzhang Yang, Myra K. Derbyshire, Roxanne A. Yamashita, Aron Marchler‐Bauer

**Affiliations:** ^1^ National Center for Biotechnology Information, National Library of Medicine National Institutes of Health Bethesda Maryland

**Keywords:** Conserved Domain Database, domain architecture, protein annotation, protein classification, protein domains, protein function, protein naming

## Abstract

The Conserved Domain Database (CDD) is a freely available resource for the annotation of sequences with the locations of conserved protein domain footprints, as well as functional sites and motifs inferred from these footprints. It includes protein domain and protein family models curated in house by CDD staff, as well as imported from a variety of other sources. The latest CDD release (v3.17, April 2019) contains more than 57,000 domain models, of which almost 15,000 were curated by CDD staff. The CDD curation effort increases coverage and provides finer‐grained classifications of common and widely distributed protein domain families, for which a wealth of functional and structural data have become available. The CDD maintains both live search capabilities and an archive of pre‐computed domain annotations for a selected subset of sequences tracked by the NCBI's Entrez protein database. These can be retrieved or computed for a single sequence using CD‐Search or in bulk using Batch CD‐Search, or computed via standalone RPS‐BLAST plus the rpsbproc software package. The CDD can be accessed via https://www.ncbi.nlm.nih.gov/Structure/cdd/cdd.shtml. The three protocols listed here describe how to perform a CD‐Search (Basic Protocol 1), a Batch CD‐Search (Basic Protocol 2), and a Standalone RPS‐BLAST and rpsbproc (Basic Protocol 3). © 2019 The Authors.

**Basic Protocol 1**: CD‐search

**Basic Protocol 2**: Batch CD‐search

**Basic Protocol 3**: Standalone RPS‐BLAST and rpsbproc

## INTRODUCTION

The Conserved Domain Database (CDD) of the National Center for Biotechnology Information (NCBI) is a collection of protein family and protein domain models. A domain is defined as a compact, discrete unit of 3D structure, typically in the range of 50 to 200 amino acids in size, and as a unit of molecular evolution that can be utilized to establish evolutionary classifications; a domain is usually associated with discrete aspects of protein function, such as enzyme activity, membrane transport, or nucleic‐acid binding, to name a few. Domain models in the CDD include many fine‐grained hierarchical classifications for selected domain families established with the help of phylogenetic analyses and manually curated by CDD staff, as well as sets of domain models imported from external high‐quality and comprehensive resources, collected as annotated multiple sequence alignments and converted into position‐specific score matrices. The current CDD collection (version 3.17) contains 57,242 total models: 14,908 models from the CDD curation effort, 35 NCBIfams (Haft et al., 2018), 1012 models from SMART v6.0 (Letunic, Doerks, & Bork, 2014), 16,709 models from Pfam v31 (Finn et al., 2016), 4873 COGs v1.0 (Tatusov et al., 2001), 10,885 NCBI Protein Clusters (Klimke et al., 2009), and 4488 models from TIGRFAM v15 (Haft et al., 2013).

**Figure 1:**
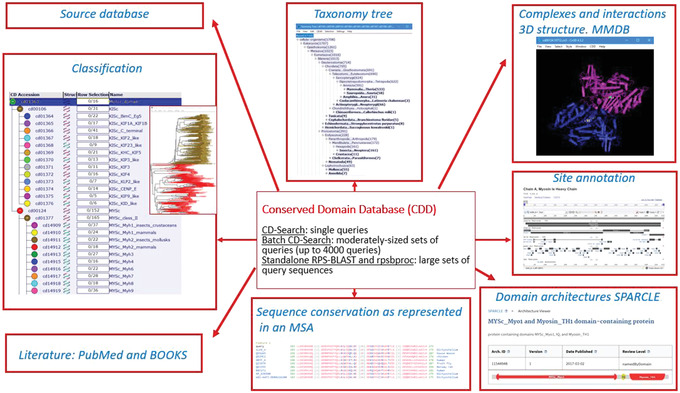
Some of the wealth of information available through the Conserved Domain Database (CDD), which includes hierarchical classifications, taxonomic information, aligned sequences, structural interaction data, domain architectures, functional site annotations, and current literature sources.

The conserved domain summary pages give access to a wealth of data associated with each domain family, including hierarchical classifications, taxonomic information, sequence alignments, structural interaction data, domain architectures, functional site annotations, and literature. Figure 1 diagrams some of the variety of information available to the user in navigating the CDD. In an effort to take advantage of these multiple types of information, the CDD uses Reverse Position‐Specific BLAST (RPS‐BLAST), also known as CD‐Search (Conserved Domain Search), in its interactive web‐based implementation to match protein sequences with domain and family models, providing a live search service for protein and nucleotide queries, as well as pre‐computed (at a pre‐set *E*‐value) domain and site annotations for the majority of protein sequences in the NCBI's Entrez system. The CDD has been integrated with several resources at the NCBI, including BLAST, Protein, and Gene, and with external collections such as InterPro (Apweiler et al., 2000; Mitchell et al., 2019; https://www.ebi.ac.uk/interpro), in order to provide a comprehensive workflow that will fit most user's needs.

You can access the CDD resource by using CD‐Search for a single nucleotide or protein sequence query, Batch CD‐Search for up to 4000 queries at a time, or standalone RPS‐BLAST plus rpsbproc running searches on your local infrastructure. You can also query Entrez (https://www.ncbi.nlm.nih.gov/cdd/) to access the CDD's domain information in the CDD resource. In Basic Protocols 1 to 3, we describe how to use each of these services so that you can customize the settings, and we outline commonly used workflows. In addition, we provide links to Help documentation (Table 1) to aid you as you navigate these pages.

**Table 1:** URLs and FTP Sites Associated with the CDD Protocols Described in This Paper

https://www.ncbi.nlm.nih.gov/Structure/cdd/cdd.shtml	CDD home page
https://www.ncbi.nlm.nih.gov/cdd	Entrez interface to the CDD
https://www.ncbi.nlm.nih.gov/Structure/cdd/wrpsb.cgi	CD‐Search Interface
https://www.ncbi.nlm.nih.gov/Structure/cdd/cdd_help.shtml	CDD Help documentation
https://www.ncbi.nlm.nih.gov/Structure/bwrpsb/bwrpsb.cgi	BATCH Web CD‐Search interface
https://www.ncbi.nlm.nih.gov/Structure/cdd/cdd_help.shtml#BatchRPSBInput	Batch CD‐Search Help documentation
https://www.ncbi.nlm.nih.gov/Structure/cdd/docs/cdd_news.html	CDD News page (for most recent domain database versions)
https://blast.ncbi.nlm.nih.gov/Blast.cgi	BLAST Homepage
https://www.ncbi.nlm.nih.gov/books/NBK279690/	BLAST® Command Line Applications User Manual
https://blast.ncbi.nlm.nih.gov/Blast.cgi?CMD=Web&PAGE_TYPE=BlastDocs&DOC_TYPE=BlastHelp	BLAST Help documentation
https://www.ncbi.nlm.nih.gov/Structure/CN3D/cn3dtut.shtml	CDD Cn3d tutorial
https://www.ncbi.nlm.nih.gov/Structure/cdtree/cdtree.shtml	CDD CDTree: protein domain hierarchy viewer and editor
https://www.ncbi.nlm.nih.gov/sparcle	Entrez interface to SPARCLE
https://www.ncbi.nlm.nih.gov/Structure/sparcle/docs/sparcle_help.html	SPARCLE Help documentation
https://www.ncbi.nlm.nih.gov/books/NBK3837/	Entrez Help documentation
https://ftp.ncbi.nih.gov/pub/mmdb/cdd	CDD FTP site; see the README file for content
https://ftp.ncbi.nih.gov/pub/mmdb/cdd/rpsbproc/	CDD rpsbproc FTP site
https://ftp.ncbi.nih.gov/pub/mmdb/cdd/rpsbproc/README	rpsbproc README file
https://ftp.ncbi.nih.gov/pub/mmdb/cdd/little_endian	CDD pre‐formatted search databases FTP site
https://ftp.ncbi.nih.gov/blast/executables/LATEST/	NCBI BLAST executables FTP site
https://ftp.ncbi.nih.gov/toolbox	NCBI C++ toolkit distribution FTP site

## Basic Protocol 1 CD‐SEARCH

**Figure 2:**
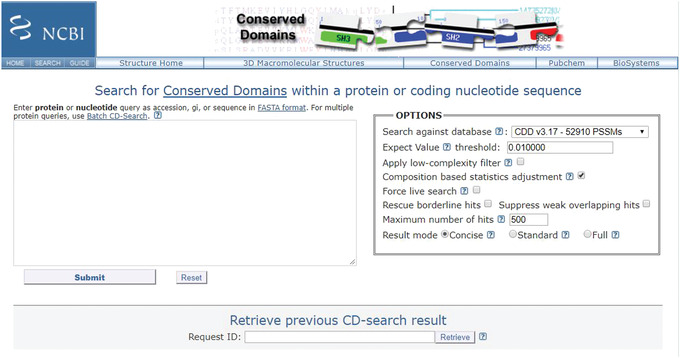
CD‐Search page using the Web server https://www.ncbi.nlm.nih.gov/Structure/cdd/wrpsb.cgi/.

The NCBI's CD‐Search service (https://www.ncbi.nlm.nih.gov/Structure/cdd/wrpsb.cgi; Figure 2) allows users to query a nucleotide or protein sequence against the CDD database via a sequence identifier or by pasting in the sequence in FASTA or raw text format. For the majority of queries provided as valid sequence identifiers, the default CD‐Search settings display results of pre‐computed RPS‐BLAST searches (storing up to 500 hits each) that were run against the entire CDD database—including CDs curated by CDD staff along with additional sources from Pfam (Finn et al., 2016), SMART (Letunic et al., 2014), KOG (Tatusov et al., 2003), COG (Tatusov et al., 2001), PRotein K(c)lusters (PRK; Klimke et al., 2009) and TIGRFAMs (Haft et al., 2013)—at an *E*‐value threshold of 0.01. The results are displayed by default in a concise format that shows the best‐scoring domain model for each region of the query sequence plus the associated domain superfamily. If a region is annotated by a model that does not score well enough to be classified as a “specific hit,” only the superfamily annotation is shown. Default CD‐Search parameters employ a score adjustment to address compositional bias, which largely abolishes the need to mask out low‐complexity regions. Basic Protocol 1 demonstrates how to identify protein domains for a single nucleotide or protein sequence.

### Necessary Resources

#### Hardware

Workstation with Internet access

#### Software

Web browser

#### Files

Protein sequence in FASTA format, accession number, or gi (GeneInfo) number

**Figure 3:**
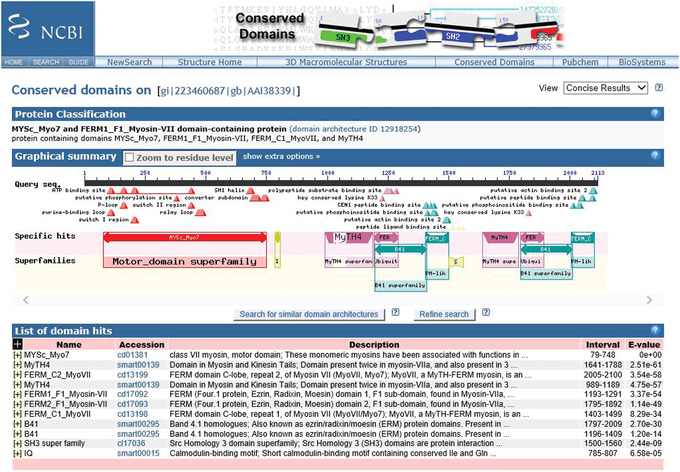
CD‐Search results for query gi 223460687 (mouse myosin VIIB protein) showing the Protein Classification, Graphical summary, and List of domain hits results.

1 Open the protein sequence search page: https://www.ncbi.nlm.nih.gov/Structure/cdd/wrpsb.cgi (see Figure 2).
2 In the text box, type the accession or gi number, or paste in the sequence of your protein or nucleotide of interest, in FASTA format.
3 To run the search with the default settings, press the **Submit** button.
4 View the results as they appear in HTML format (see Figure 3).

**Figure 4:**
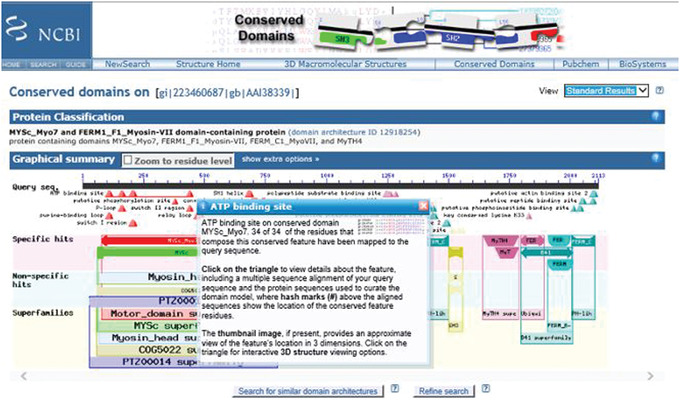
CD‐Search results for query gi 223460687 (mouse myosin VIIB protein) showing mouse‐over pop‐up of the ATP‐binding site annotation.

5 Select the scope of your graphical summary display by going to the top right‐hand corner of the display and using the **View** pulldown menu to select either **Concise Results**, **Standard Results**, or **Full Results**.
  The display default is a view of the **Concise Results**, as shown in Figure 3. See to the Guidelines for Understanding Results section of this article for an explanation of the different views.
6 Scroll over the annotations marked by triangles under the Query sequence in the Graphical Summary to reveal a pop‐up window with information about a functional feature mapped to the query sequence via a domain hit. The pop‐up window links to a CD summary page, which shows the multiple sequence alignment of protein sequences used to curate the model, annotated with hash marks denoting the location of the conserved feature residues, and providing the option to examine evidence supporting the feature.
  An example of a result for an annotated site is shown in Figure 4.

**Figure 5:**
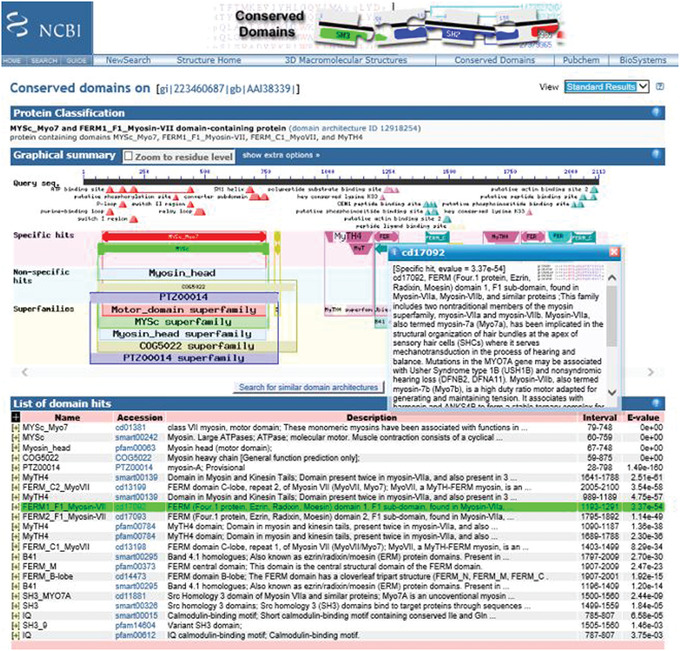
CD‐Search results for query gi 223460687 (mouse myosin VIIB protein) showing mouse‐over pop‐up of the FERM domain.

7 Scroll over the cartoon of the CD domain to reveal a pop‐up panel showing the *E*‐value, accession ID, name, and description. This also highlights the corresponding domain hit (shown in green) in the **List of domain hits**.
  An example of this type of result for a CD domain is shown in Figure 5.

**Figure 6:**
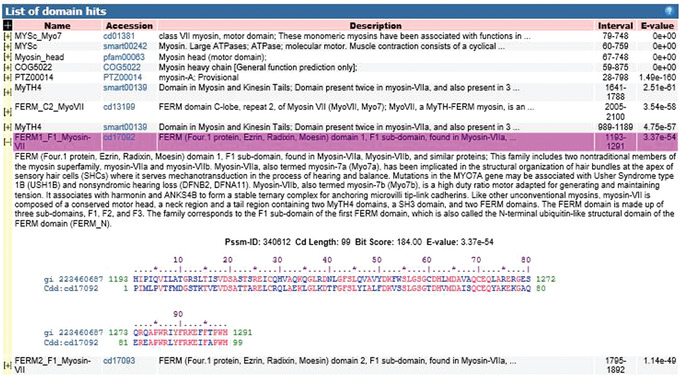
CD‐Search results for query gi 223460687 (mouse myosin VIIB protein) showing expanded FERM domain with domain definition and query alignment to CD.

8 Click on the plus [+] in the **List of domain hits** to see how your query is aligned with the domain model.
  An example of an expanded domain hit for a CD domain is shown in Figure 6.

**Figure 7:**
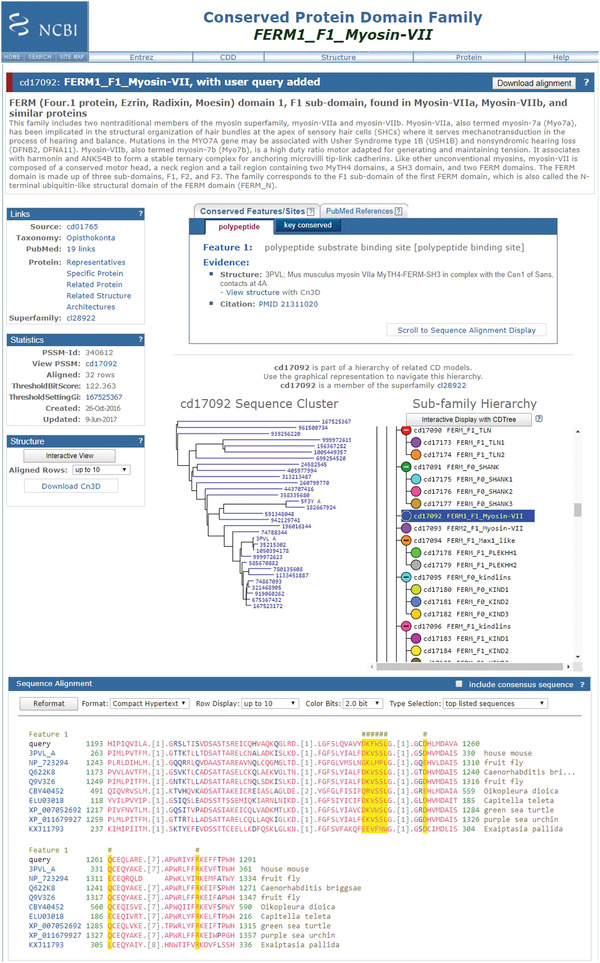
CD‐Search results summary page for FERM domain with domain definition and user query added to the CD multiple sequence alignment.

**Figure 8:**
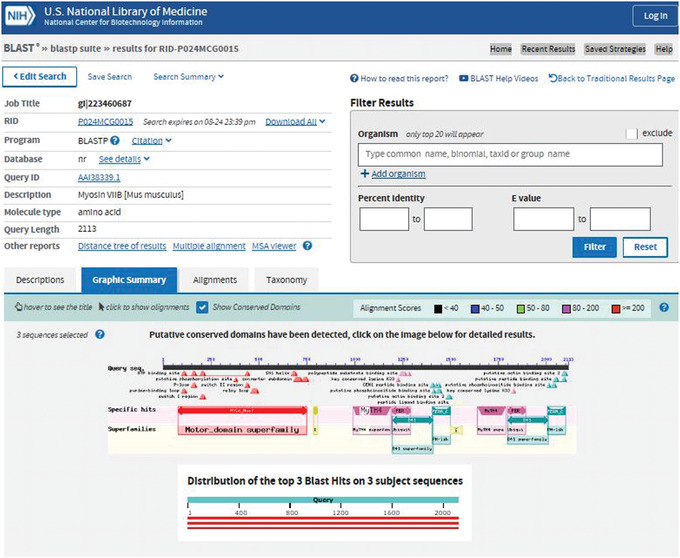
CD‐Search results page for the query gi 223460687 (mouse myosin VIIB protein) found under the **Graphic Summary** tab in BLAST.

9 To launch and view the CD summary page on your domain of interest, click on the CD link in the List of Domain Hits, and click on the cartoon “bubble” of the CD of interest or on the symbols (triangles) indicating the location of feature annotations. Invoking the CD summary pages via links from the Graphical Summary will result in your query imbedded into the sequence alignment on the CD summary page.
  An example of a CD domain summary page is shown in Figure 7.
  CD‐Search results can also be accessed through the protein BLAST results pages, because CD‐Searches are also run during protein BLAST searches. In the recently revised BLAST results pages, adopted in August 2019, CD‐Search results appear under the **Graphic Summary** tab. If conserved domain hits are detected on the query sequence, you will see the message “Putative conserved domains have been detected”; clicking on the image below this message will take you to the familiar CD‐Search results page for your query.
  An example of a BLAST search of gi 223460687 (mouse myosin VIIB protein) showing the CD‐Search result is shown in Figure 8.

## Basic Protocol 2 BATCH CD‐SEARCH

Use Batch CD‐Search (https://www.ncbi.nlm.nih.gov/Structure/bwrpsb/bwrpsb.cgi) to compute and retrieve domain annotations for a batch of protein queries. Basic Protocol 2 demonstrates how to identify protein domains for a batch of protein queries up to 4000 sequences. The limits may be adapted in the future due to the high peak usage of this shared resource.

### Necessary Resources

#### Hardware

Workstation with Internet access

#### Software

Web browser

#### Files

**Figure 9:**
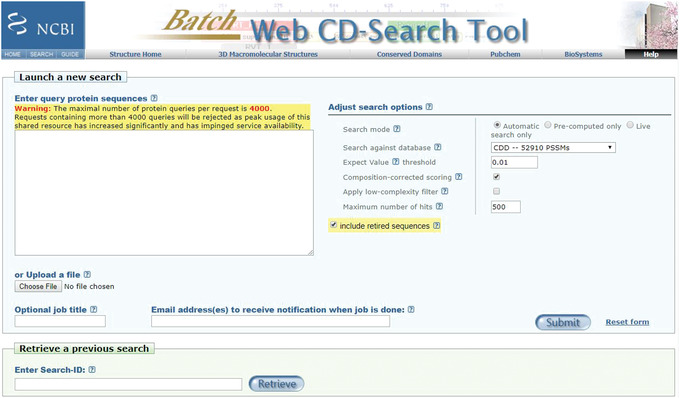
Batch CD‐Search page using the Web server https://www.ncbi.nlm.nih.gov/Structure/bwrpsb/bwrpsb.cgi/.

A list of protein sequences in FASTA format, raw text format (lines of sequence data, without the FASTA definition line), accession number, or gi (GeneInfo) number, and separated by line breaks; different query types can be mixed in a single Batch CD‐Search (for more detailed information on input format, consult the Batch CD‐Search Help documentation, at https://www.ncbi.nlm.nih.gov/Structure/cdd/cdd_help.shtml#BatchRPSBInput; we provide a test set of 1348 sequences named “MYCs Myosin motor domain cd00124 sequences” in the Supplementary Materials)
The Batch CD‐Search page (https://www.ncbi.nlm.nih.gov/Structure/bwrpsb/bwrpsb.cgi; Figure 9)

### Run the search

**Figure 10:**
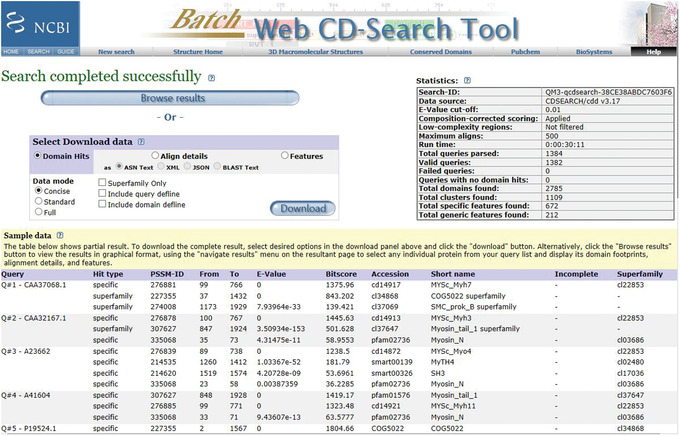
Batch CD‐Search. Preliminary results web page for the myosin motor domain test set.

1 Enter your list of query proteins directly into the text box, or upload the list as a text file.
  Unlike CD‐Search (Basic Protocol 1), Batch CD‐Search does not accept nucleotide queries.
2 Add a title to your job in the **Optional job title** text box.
  This title will appear in the subject line of the notification email.
3 Input your email address(s) in the **Email address(es)** text box, so that you will be notified when the job is complete.
  If you enter multiple email addresses, separate these with commas.
4 Run Batch CD‐Search by pressing the “**Submit**” button or hitting Enter.
  Batch CD‐Search will run with the default settings.
5 View the Preliminary Results. If the search has been successful, a preliminary web page will be returned displaying the message “Search completed successfully” and with **Sample data**.
  The sample data include an indication of whether the domain hit is a specific or a superfamily hit, the PSSM‐ID, the from‐to domain intervals, the E‐value, BitScore, the domain accession, the domain short names, and the CDD Superfamily cluster. An example of preliminary web page output obtained using the myosin motor domain test set provided is shown in Figure 10.

6 Save the **Search‐ID**: Save the complete **Search ID** string found at the top of the **Statistics** box to access the complete results (master data structure) for up to 2 days after the search is first run.
7 Browse the complete results (master data structure).
  You have the option to **Browse results** and/or **Download data**.

### Browse results

**Figure 11:**
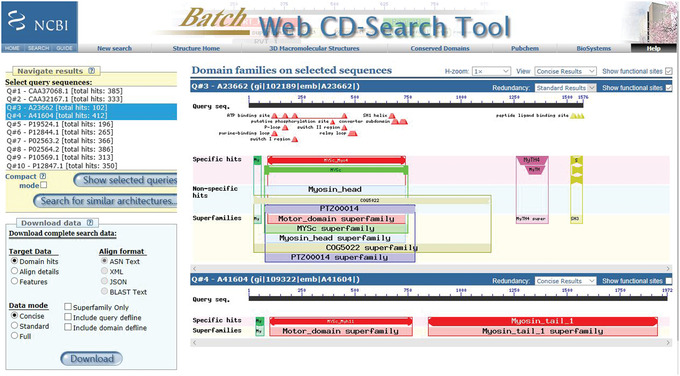
BLAST CD‐Search: Browse results output for the myosin motor domain test set: showing query Q#3 with **Standard results** and with “Show functional sites” selected, and query Q#4 with **Concise results** and with “Show functional sites” unselected.

8 Press the **Browse results** button on the Preliminary Results web page.
  This launches a page similar to the one shown in Figure 11 except that only the first query protein is selected and its CD‐Search result shown.

**Figure 12:**
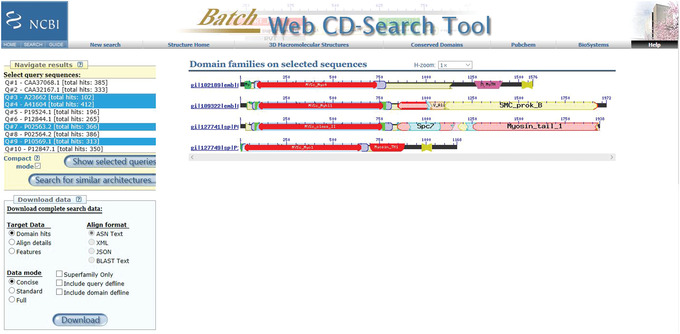
BLAST CD‐Search: Browse results **Compact mode** output for the myosin motor domain test set, showing the domain architecture for the queries Q#3, #4, #7, and #9 each on a single line.

9 Browse and compare multiple results. In the **Navigate Results** panel, select multiple query sequences by holding down the keyboard Ctrl key and using the keyboard arrow keys to scroll through the query list. Then press the **Show selected queries** button to display your selections.
  The default redundancy is to view Concise results and show functional sites. You can customize both redundancy (to view Concise, Standard, or Full results) and whether to show functional sites, either on an individual protein basis or for the complete set. An example of this for the myosin motor domain test set is shown in Figure 11.
10 To view results in Compact mode, in the **Navigate Results** panel, check the **Compact Mode** box, and then press the **Show selected queries** button.
  An example of Compact‐view output for the myosin motor domain test set is shown in Figure 12.
  In this mode you can compare, at a glance, the domain architectures and different domains of your selected sequences.

**Figure 13:**
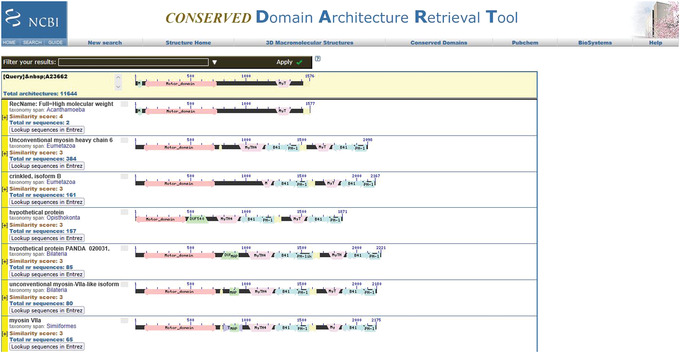
BLAST CD‐Search: “Search for similar architectures” output for the myosin motor domain test set for query Q#3. Each hit represents a unique domain architecture that contains one or more domains of query Q#3.

11 To search for similar architectures, in the **Navigate Results** panel, select a query sequence, and then press the **Search for similar architectures** button.
  An example **Search for similar architecture** (CDART; Geer et al., 2002) output for the myosin motor domain test set is shown in Figure 13.
**Search for similar architectures** launches the Conserved Domain Architecture Retrieval tool (CDART) program, which looks for domain architectures (DA; i.e., sequential order of conserved domains) that are similar at the domain superfamily level to that of your selected protein. Each hit represents a unique DA that contains one or more domains of the selected protein. CDART groups together proteins that share the same DA. Clicking on the (+) beside a CDART hit opens a list of representative, nonredundant (nr) sequences in that group.

### Download the data

**Figure 14:**
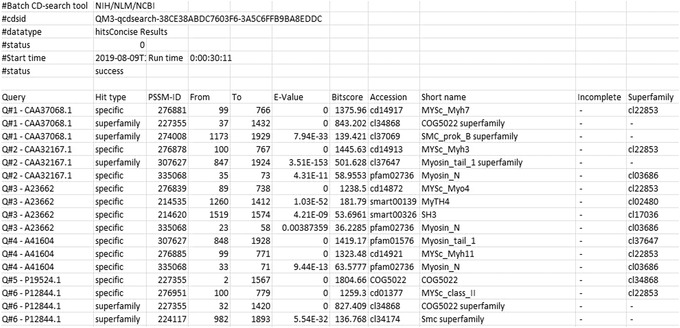
Batch CD‐Search: Downloading Domain hit results with the default parameters **Target data**: Domain Hits and **Data mode**: Concise for the myosin motor domain test set.

12 From the **Browse results page**, you can download three categories of **Target Data**: Domain hits, Align details, and Features.
  You can also do this from the Preliminary web page (Figure 10).
13 To download Domain Hit Data, on the **Browse results page**, select the **Download data** panel, with the default setting (**Target data**: Domain Hits and **Data mode**: Concise), and press the **Download** button.
  An example Domain Hits output for the myosin motor domain test set is shown in Figure 14 (the output ASN text file was copied and pasted into Excel).
  The Domain hits output is a tab‐delimited table that returns CDD models that have statistically significant hits to the protein sequences in your query list. Additional options are available when downloading these data. For example, to check the Superfamily only, include the query **defline** and include the domain **defline boxes**, as well as defining which level of DataMode/Redundancy you prefer: Concise, Standard, or Full. The Domain Hits output includes the Query (Q#N, where N is the ordinate number of the query sequence in your original input list, followed by either the unique sequence identifier, the first 15 characters of the FASTA defline, or the first 15 amino acids of the bare sequence data), the Hit Type (specific hits or superfamily hit), the domain from‐to interval, the E‐value, the BitScore (defined at https://www.ncbi.nlm.nih.gov/books/NBK21106/def‐item/app8/) for comparing alignment scores between different CD‐Searches, the domain's CDD accession number, and the short name.

**Figure 15:**
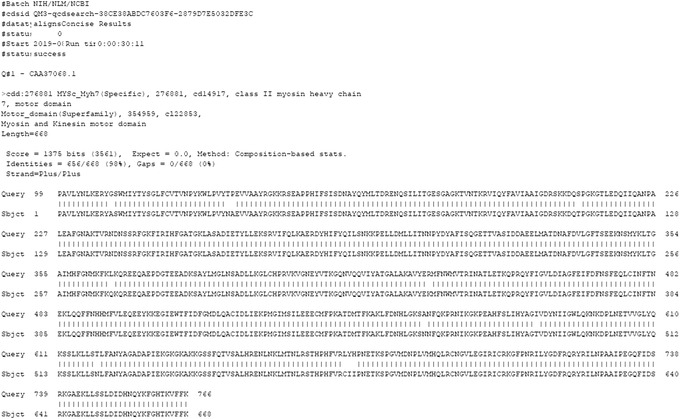
Batch CD‐Search: Downloading Align Details results with the parameters **Target Data**: Align details, **Align format**: BLAST Text, and **Data mode**: Concise, for the myosin motor domain test set (only the output for query Q#1 is shown).

**Figure 16:**
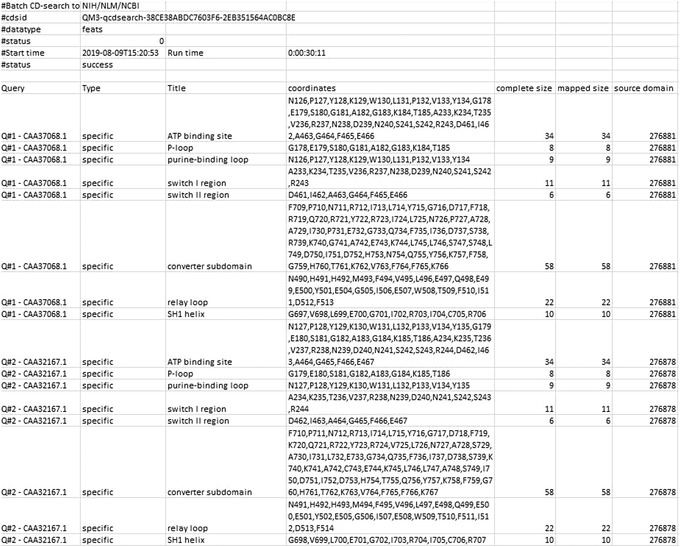
Batch CD‐Search: Downloading Features results with the parameters **Target data**: Features, **Align format**: ASN Text, and **Data mode**: Concise, for the myosin motor domain test set (only the output for queries Q#1 and Q#2 are shown).

14 To download Alignment details data, from the **Browse results page**, in the **Download data** panel, with appropriate settings (**Target Data**: Align details; **Align format**: BLAST text; and **Data mode**: Concise), press the **Download** button.
  An example Align details output for the myosin motor domain test set is shown in Figure 15 (the output BLAST text file).
  The Align details output is in a familiar BLAST format, with pairwise alignment details between the query sequence and the consensus sequence from each domain model or superfamily that has a hit. Exact matches are marked by a pipe symbol “I” between the query and consensus sequence. In addition to BLAST Text, you have the option to download **Align format** as ASN.1 (Abstract Syntax Notation One) text, XML (Extensible Markup Language), or JSON (JavaScript Object Notation).
  To download Features Data, open the **Download data** panel in the **Browse results page**, with appropriate settings (**Target data**: Features, **Align format**: ASN Text, and **Data mode**: Concise), and press the **Download button**.
  An example of Features output for the myosin motor domain test set is shown in Figure 16 (the output ASN text file was copied and pasted into Excel).
  The Features output is a tab‐delimited table that lists conserved features annotated on the domain models as mapped to the query; it includes the Query number, the Type (categorization as specific or nonspecific hit), the Title of the feature, the Coordinates (single‐letter amino acid codes and their positions on the query sequence), the complete size (the total number of residues in the conserved feature that have been annotated on the domain model), the mapped size (the number of residues in the query protein that match residues in the feature that was annotated on the domain model), and the source domain (the PSSM ID of the domain model on which the feature was annotated).

## Basic Protocol 3 STANDALONE RPS‐BLAST AND rpsbproc

Use Standalone RPS‐BLAST and rpsbproc (https://ftp.ncbi.nih.gov/pub/mmdb/cdd/rpsbproc/e) to compute and retrieve domain annotation programmatically. Basic Protocol 3 demonstrates how to identify protein domains for a batch of protein queries of greater than 4000.

### Necessary Resources

#### Hardware

An internet‐connected Linux, Windows, or Mac workstation

#### Software

Web browser, for downloading files from FTP site
The tar utility, to extract files from compressed archive files: A built‐in utility for the Linux, Windows, and Mac platforms, found in Shell (Linux), Windows Command Processor (Windows), and Terminal (Mac), respectively
The gzip utility, required to decompress files: For the Linux and Mac platforms, commonly a built‐in utility by default; for the Windows platform, the specified software, including 7‐Zip, WinZip, and others, can be used
The curl utility, for downloading files from FTP site (optional): For the Linux platform, commonly installed by default; for Windows and Mac platforms, can be downloaded from https://curl.haxx.se/download.html and installed manually
Specific FTP software, for downloading files from FTP site more efficiently (optional): e.g., FileZilla

#### Files

Input queries in FASTA format: i.e., protein or nucleotide sequences

### Preliminary Steps

Detailed instructions on how to retrieve the RPS‐BLAST executable and rpsbproc utility and run them locally can be found in the rpsbproc README file at the CDD FTP site (https://ftp.ncbi.nih.gov/pub/mmdb/cdd/rpsbproc/README).

**Figure 17:**
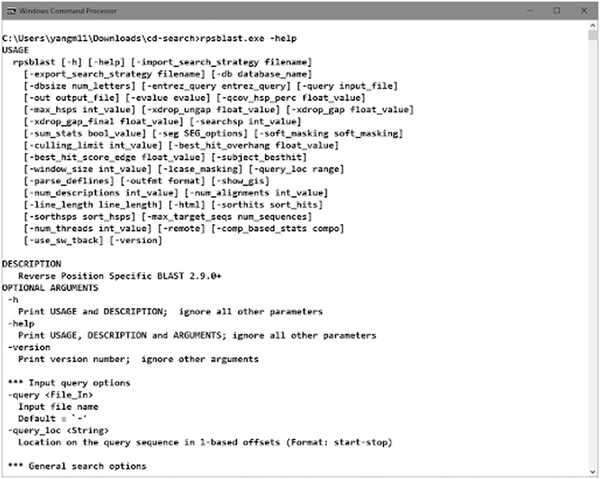
Run command to view the help documentation of the rpsblast executable.

The standalone RPS‐BLAST packaged with the pre‐built BLAST executables (“rpsblast” for protein queries and “rpstblastn” for nucleotide queries) is available at the NCBI BLAST FTP site and as part of the NCBI C++ toolkit distribution. Detailed documentation for BLAST at NCBI, including RPS‐BLAST, can be found in BLAST^®^ Command Line Applications User Manual (https://www.ncbi.nlm.nih.gov/books/NBK279690/). Run the command rpsblast with argument “‐help” to check the usage information (Figure 17).

For each query sequence, standalone RPS‐BLAST lists the conserved domain models that scored below a certain *E*‐value threshold (by default set to 10), sorted by *E*‐value. For each hit, information such as the conserved domain's PSSMID, a set of scores (*E*‐value, BitScore, etc.), and the sequence alignment between the conserved domain and the query sequence can be returned. In order to run the rpsbproc utility, the output file generated by RPS‐BLAST executables needs to be stored in ASN.1 format, using “.asn” as the filename extension.

**Figure 18:**
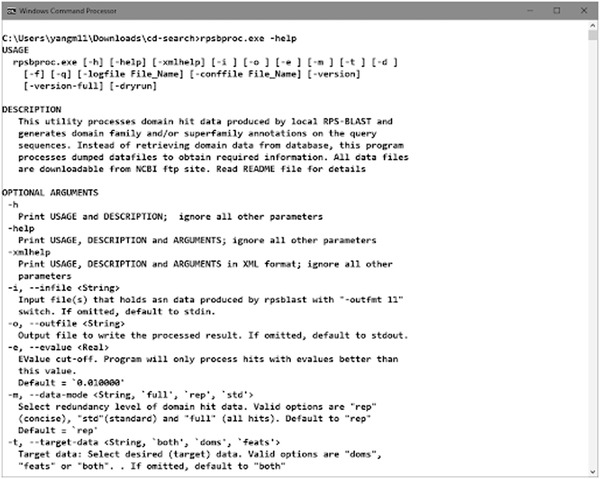
Run command to view the help documentation of the rpsbproc executable.

The rpsbproc command line utility is an addition to the standalone version of RPS‐BLAST. It post‐processes the RPS‐BLAST output to give a compact and nonredundant view of the search results (such as would be returned by the Batch CD‐Search). rpsbproc reads the output of rpsblast/rpstblastn and fills in domain superfamily and functional site information, as well as structural motifs, for each region of the sequence. It then re‐sorts the hits and calculates a set of nonredundant representative hits. The result is presented in a tab‐delimited flat file and can be looked at either programmatically or manually. Run rpsbproc command with argument “‐help” to check the usage information (Figure 18).

To run RPS‐BLAST locally and use rpsbproc to process the output, you must first collect the applications needed. You can download the pre‐built rpsblast, rpstblastn, and rpsbproc binaries from the NCBI FTP site, which are directly executable on Windows and Linux platforms, with no complex installation required. For those who need (or desire) to build these utilities locally, you can download the source code tarballs from the NCBI FTP site. Please note that these programs are NCBI C++ toolkit applications and require the NCBI C++ toolkit to build. Please follow the README file to build these utilities locally. For Linux and Mac users, please refer to the rpsbproc README file for detailed instruction to run standalone RPS‐BLAST and rpsbproc utility. Below are step‐by‐step instructions for running these executables on a Windows platform.

### Procedure

**Figure 19:**
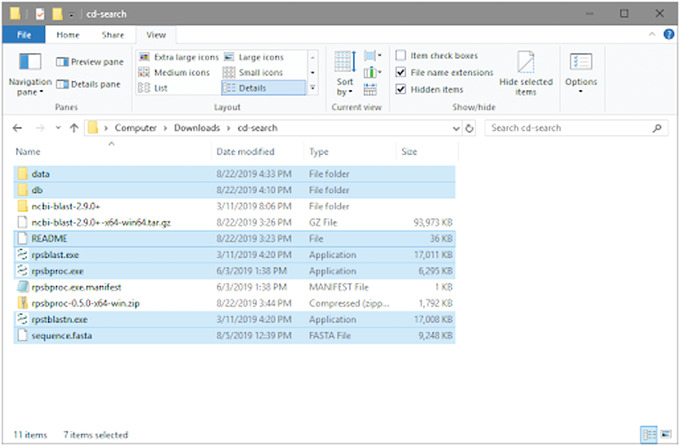
CD‐Search Folder with applications required to run standalone RPS‐BLAST and rpsbproc. The items shown with highlighted backgrounds are required to run RPS‐BLAST and rpsbproc.

1 Download the rpsbproc README file (https://ftp.ncbi.nih.gov/pub/mmdb/cdd/rpsbproc/README) to your project folder for reference in the following steps.
  An example of the project folder labeled cd‐search is shown in Figure 19.

**Figure 20:**
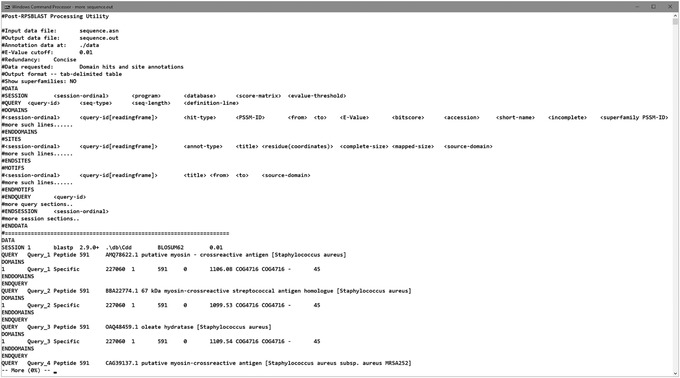
Results from running the rpsbproc utility on myosin sequences.

2 Retrieve the RPS‐BLAST executable by downloading the RPS‐BLAST executable (ncbi‐blast‐2.9.0+‐x64‐win64.tar.gz) to the project folder from the NCBI BLAST FTP site (https://ftp.ncbi.nih.gov/blast/executables/LATEST/). Then, open the Windows Command Processor (cmd.exe) and navigate to the project folder to run the command below to uncompress the downloaded file, which creates a folder named ncbi‐blast‐2.9.0+ in the project folder.
tar ‐zxf "ncbi‐blast‐2.9.0+‐x64‐win64.tar.gz"
  Navigate to the bin sun‐folder in ncbi‐blast‐2.9.0+, and copy the executables rpsblast.exe and rpstblastn.exe to the project folder.
3 Retrieve the rpsbproc executable by downloading the executable (rpsbproc‐0.5.0‐x64‐win.zip) to the project folder from the NCBI CDD FTP site (https://ftp.ncbi.nih.gov/pub/mmdb/cdd/rpsbproc/). In the Windows Command Processor, navigate to the project folder and run the command below to uncompress the zip‐file downloaded.
tar ‐zxf rpsbproc‐0.5.0‐x64‐win.zip
  Now you have rpsbproc.exe and rpsbproc.exe.manifest files in the project folder.
4 Create the search database for RPS‐BLAST by downloading the preformatted search database (files) from the CDD FTP site (https://ftp.ncbi.nih.gov/pub/mmdb/cdd/little_endian/) to the folder named db under the project folder. Uncompress the files separately in the current db directory using the commands below:
tar ‐zxf Cdd_LE.tar.gz
tar ‐zxf Cdd_NCBI_LE.tar.gz
tar ‐zxf Cog_LE.tar.gz
tar ‐zxf Kog_LE.tar.gz
tar ‐zxf Pfam_LE.tar.gz
tar ‐zxf Prk_LE.tar.gz
tar ‐zxf Smart_LE.tar.gz
tar ‐zxf Tigr_LE.tar.gz
5 Create the data folder by downloading the domain‐annotation files (listed below) from the CDD FTP site (https://ftp.ncbi.nih.gov/pub/mmdb/cdd/) to the data folder.
bitscore_specific.txt
cddannot.dat.gz
cddannot_generic.dat.gz
cddid.tbl.gz
cdtrack.txt
family_superfamily_links
  The three files with “.gz” suffix can be unzipped with software such as 7‐Zip in Windows. Make sure you choose “Extract here” to unzip the file into the current data folder.
6 Put your FASTA file containing query sequences into the project folder. sequence.fasta was used in this example.
  An example of how your folder contents should look is shown in Figure 19.
7 Run RPS‐BLAST by opening the Windows Command Processor (cmd.exe). Navigate to the project folder and run RPS‐BLAST using the command below. Backslashes are used because this command is run on a Windows command processor.
rpsblast.exe ‐query sequence.fasta ‐db .\db\Cdd ‐evalue 0.01 ‐outfmt 11 ‐out sequence.asn
8 Run the rpsbproc executable using the command below to annotate the results generated by RPS‐BLAST.
rpsbproc.exe ‐i sequence.asn ‐o sequence.out ‐e 0.01 ‐m re
9 View the results. The output file has a tab‐delimited format and can be opened with WordPad, Excel, or similar editors.
  An example of what your output file should look like is shown in Figure 20.

## GUIDELINES FOR UNDERSTANDING RESULTS

### Basic Protocol 1

CD‐Search allows users to query a nucleotide or protein sequence against the CDD database via its accession number or gi number, or by pasting in the sequence in FASTA or raw text format using RPS‐BLAST. The CDD database includes CDs curated in house by the NCBI along with additional sources from Pfam, SMART, KOG, COG, PRK, and TIGRFAM. The results are displayed by default in a concise format that shows the best‐scoring domain model for each region of the query together with the corresponding domain superfamily, and the superfamily annotation only if the hit was not strong enough to be classified as specific (high confidence).

The resulting CD‐Search results display contains three sections: Protein classification, Graphical summary, and List of domain hits for the query. At the top, above the protein classification section, it shows the query as well as the view that is currently being used (Concise Results, Standard Results, or Full Results). The CD Summary page can be launched from either the Graphical summary or the List of domain hits and contains detailed information about your domain of interest.

#### Section 1: Protein Classification

The Protein classification section displays a suggested name for the query protein, a label that may specify a suggested function, and a link to the SPARCLE (Subfamily Protein Architecture Labeling Engine; Marchler‐Bauer et al., 2017) classification (Figure 3).

#### Section 2: Graphical Summary

The Graphical summary shows the domain hits and annotated features. Feature annotations are denoted by triangles colored the same as the domains they correspond to. The **results mode** display can be chosen in the CD Search panel or changed after the search is run by changing the selection in the **View panel** (see Figure 3). The **standard display** format shows the best‐scoring domain model from each data source (best Pfam hit, best COGs hit, etc.). The **full display format** shows all matching domain models identified by RPS‐BLAST for each region of the query sequence and can be very redundant. The display can be customized to hide the display of site annotation features by selecting the **show extra options** and deselecting the **Show site features**, as well as magnifying the display using the **Horizontal zoom** and the **Zoom to residue level** selections.

Hovering over the triangles in the site features triggers a pop‐up window with information on the number of feature residues that map to the query sequence. Clicking on the triangle takes you to the CD summary page, where your query is embedded into the CD alignment with the residues involved in the site features highlighted and marked with hash marks (#).

Hovering over the domains triggers a pop‐up window with a description of the domain and highlights the corresponding row in the **List of domain hits** panel. Clicking on the domain graphic also takes you to the CDD page with your query embedded into the CD alignment.

#### Section 3: List of Domain Hits

The **List of domain hits** lists the conserved domains identified on the query sequence. For each conserved domain identified, it displays its short name, its accession number, a description of the domain, the interval on the query that is covered by the domain footprint, and the *E*‐value. Clicking on the (+) next to each name reveals the full description of the domain and shows the alignment of the query sequence to a representative (consensus) sequence of the domain model, together with the numerical domain model identifier (PSSM‐ID) and the alignment bit score. Click on the domain model's accession number to view the multiple sequence alignments of the proteins used to develop the corresponding domain model. Note that your query sequence is not embedded in this version of the CD summary page.

If a live search was performed, the **BLAST Request ID (RID)** is shown at the bottom of the **Standard** and **Full** displays and allows you to retrieve the search results using the RID anytime within the 36 hr following the search, without having to re‐execute it.

To change the search settings, click on the **Refine Search** button (which will retain your query) or select **New Search** from the selection bar immediately below the logo at the top of the page. Go to the **OPTIONS** panel. Use the **Search against database** option pulldown to select a specific database. Change the *E*‐value to stricter or more permissive by changing the value in the **Expect Value threshold** option. If you would like to mask out compositionally biased regions, check **Apply low‐complexity filter** (the graphical display of results will then highlight masked‐out regions on the query). **Composition based statistics adjustment**, which is selected by default, abolishes the need to mask out compositionally biased regions in query sequences, for the most part. Keep both the **Composition based statistics adjustment** and the **Apply low‐complexity filter** options on at the same time to filter out some false positives that may still slip through the cracks of the composition‐correction, or click both of them off to find more distant relatives for compositionally biased queries. To perform a live search, check the **Force live search** box (it will be checked if you choose settings different from the CD‐Search default). You can also **Rescue borderline hits** and **Suppress weak overlapping hits** by selecting the appropriate boxes (Derbyshire, Lanczycki, Bryant, & Marchler‐Bauer, 2012).

#### CD Summary Page

At the top of the CD summary page, you will see the CD accession number and a description of the CD. Below this you may see a box with a tab for **Conserved Features/Sites**, which contains the name(s) of the annotated site(s), evidence of various types (structure evidence, PMID references to literature, and free‐text comments), and a tab for **PubMed References** that lists relevant articles about the specific domain or protein family and more generic reviews of the wider superfamily. Annotation selections are highlighted in the **Sequence Alignment** panel and noted by hash marks at the very bottom of the page that show how the query sequence is aligned with respect to the CD sequences.

Below the Conserved Features/Sites and PubMed References panel, there is a **Sequence Cluster tree** of the CD that matched your query. If the domain is part of a hierarchical classification, you will also see a tree‐like representation of that hierarchy, with the CD that matched your query highlighted with a dark blue background, as shown in Figure 7.

Click on the **Interactive Display** with the CDTree button after selecting to download the selected CD or the entire hierarchy for viewing and further analysis with the CDTree software package (https://www.ncbi.nlm.nih.gov/Structure/cdtree/cdtreeInstall.shtml).

The right hand side of the CD summary page contains information blocks titled **Links** (Source, Taxonomy, PubMed, Protein, and Superfamily), **Statistics** (PSSM‐Id, View PSSM, Aligned, ThresholdBitScore, ThresholdSettingGi, Created and Update dates), and **Structure information**, where you can **download Cn3d**, a molecular structure and multiple sequence alignment viewer (https://www.ncbi.nlm.nih.gov/Structure/CN3D/cn3d.shtml) to visualize and manipulate the sequence and structure alignment for your query in the context of its CD hit.

Select the **Interactive View** after setting the number of aligned rows that you would like displayed in Cn3D. Upon launching, it displays three panels: a CDD Descriptive Items panel that shows some of the information found on the CD summary page (name, description, annotation, and references), a visualization window that shows the model's 3D structures if present, and a multiple sequence alignment window containing the query sequence embedded in the CD alignment.

### Basic Protocol 2

Basic Protocol 2 can run CD‐Search on a batch of up to 4000 proteins in a single request and accessed via a web service or programmatically. A single Batch CD‐Search returns annotation data in a tabular form suitable for further processing, including domain hit from‐to intervals, *E*‐values and scores, domain model names and accessions, and the positions of functional sites such as catalytic residues, binding sites, and motifs. A wealth of information on your protein collection is returned in a single search.

When **Browsing** results, please find help for interpreting the graphical results for each individual protein in Basic Protocol 1 (single CD‐Search): Guidelines for understanding the results: Graphical summary.

Basic Protocol 2 as described is run in the default mode. There are many options you may opt to modify.

The default search mode described is the **automatic search**, which either runs a live RPS‐BLAST search or retrieves precalculated results for each single item on the list depending on its sequence format. For most query sequences specified via sequence identifiers, precalculated RPS‐BLAST results are available and will be retrieved; if no results are available, a live search will be executed. For queries entered as FASTA or raw sequence, live searches will be run. You may opt for a **Live search only** mode, which runs a live RPS‐BLAST search for every item on the query list, or a **Pre‐computed only** mode, which only retrieves a precalculated RPS‐BLAST result where available but will ignore other queries.

The default mode (**automatic** search) runs against the complete CDD database (i.e., includes the CDD in‐house‐curated models and those from external sources including Pfam, SMART, KOG, COG, PRK, and TIGRFAMs) at an *E*‐value threshold of 0.01. The **Search against database** pull‐down menu provides the option to limit your search to only the NCBI in‐house‐curated subset, or to any one of the other databases included in the CDD. The current version number for each database can be found at https://www.ncbi.nlm.nih.gov/Structure/cdd/docs/cdd_news.html. You also have the option to enter a different *E*‐value threshold.

The default mode includes obsolete or preliminary sequences, and the output flags these as non‐current. You may opt to exclude these by unchecking its box on the search page.

In the default mode, the **Apply a low‐complexity filter** is turned off, but you may elect to turn it ON by checking its box on the search page to mask compositionally biased regions in the query protein sequences.

In the default mode, the **Maximum number of hits** returned is 500, as the number of expected domain hits is small for an average protein.

As the number of queries per Batch CD‐Search run is limited, and as the maximum throughput of the resource is restricted by the number of servers available on the back end, you may opt to run searches locally on your own hardware. Basic Protocol 3 describes how to run standalone RPS‐BLAST plus the rpsbproc command‐line utility. It returns annotation in a tabular format similar to that of Batch CD‐Search, suitable for further processing, and allows you to run RPS‐BLAST with customized PSSM subsets.

### Basic Protocol 3

Basic Protocol 3 runs standalone RPS‐BLAST and rpsbproc to process a large amount of protein/nucleotide sequences, and returns annotation data similar to that of batch CD‐Search (Basic Protocol 2), which include domain hits, site annotations, and structural motifs. Additionally, it allows you the option of running RPS‐BLAST locally on your own machine and, optionally, with your own PSSM subsets.

The output file generated by the rpsbproc utility comprises two sections. The first section displays the program information, parameters used for data processing, and a “template” explaining the format and content of each column of the data table. All the lines in this section start with a “#” character so that programs can treat them as “comment” lines that can be safely ignored.

**Figure 21:**
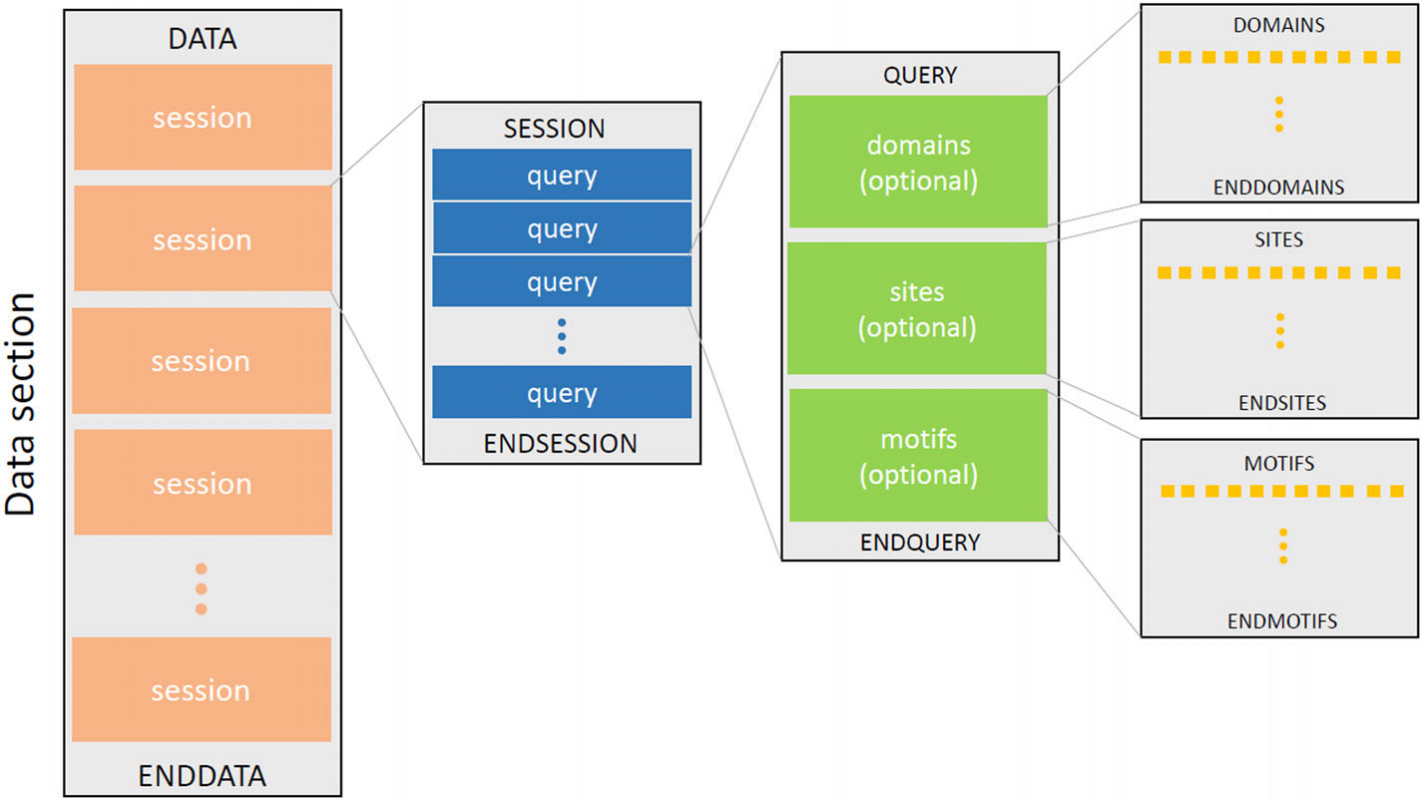
Illustration of the structure of the data section.

The second section, known as the data section, contains the real data intended to be programmatically processed. All columns are delimited with a tab character (“\t”). The data section always starts with a DATA token and ends with an ENDDATA token. In between, there can be several sessions, each of which start with a SESSION token and end with an ENDSESSION token. Each session is given an ordinal and unique number, which is known as the session ID. Each session is composed of queries, which are unit blocks of sessions. Every single query block contains three optional sections, namely domains, sites, and motifs. The full structure of the data section is illustrated in Figure 21. The domains, sites, and motifs sections contain rows of values, corresponding to the column names defined in the first section of the rpsbproc output file. In the domain section, for example, each row represents a domain hit, including the following information: session ID; query ID; hit type; PSSM ID; start position; end position; *E*‐value; bit score, accession; short name; and whether the alignment is incomplete on the N terminus, C terminus, or both; and superfamily PSSM ID (similar to the data shown in Figure 14).

## COMMENTARY

### Background Information

A protein domain is typically associated with a function, such as enzyme catalysis or nucleic‐acid binding, and is a unit of molecular evolution; via comparative sequence analysis, protein domain sequences can be organized into an evolutionary classification. The CDD's curated domain collections are often classified to a very fine‐grained level with the help of available 3D structure to guide multiple sequence alignments, and are manually annotated with functional sites using evidence from 3D structure and other information, including the published literature. Having information about a protein's domain(s) can give you (the user) a wealth of information about your protein of interest. In the cases of unclassified or novel proteins, this domain information provides vital clues to protein function, and often domain annotation is the only available hint toward molecular and cellular function for novel uncharacterized proteins.

In addition to results from the in‐house curation effort, the CDD contains domain models from external sources such as Pfam. Agreement between annotations from two or more resources provides users confidence about the domains identified, whereas disagreements between them—which may be as trivial as different domain boundary definitions, or more serious in the case where different functional domains are identified for the same region of a query—may indicate that results should be interpreted with caution.

The three CD‐Search protocols described in this paper outline methods for users to submit queries of a single protein or in batches of very large numbers of proteins. The results from these searches—such as domain model identification and accessions, domain footprints (from‐to intervals) on the query, *E*‐values and scores, and the locations of functional sites and interactions—can for larger numbers of queries be returned in a tabular form suitable for further processing.

The CDD was first described in the literature in 2002 (Marchler‐Bauer et al., 2002). Version v1.54 then contained 3693 models, including contributions from the CDD's in‐house curation, Pfam, and SMART. CDD v3.17 (April 3, 2019) contains 57,242 total models from all Source databases, 14,908 of them from the CDD curation effort.

### Critical Parameters

The current limitation of 4000 sequences for Batch CD‐Search was imposed by the CDD due to high peak usage of this shared resource; you will be alerted to any future changes to this upper limit on the Batch CD‐Search page.

To demonstrate the various CD‐Searches for Basic Protocols 1 to 3, we have provided test sets. The Batch CD search test set was derived from an in‐house‐curated MYSc myosin motor domain intermediate model (cd00124) of the cd01353 Motor Domain hierarchy, which was released on February 5, 2015. The Standalone RPS‐Blast and rpsbproc test set is a FASTA file that contains all protein records returned by searching NCBI Protein database with the search term myosin AND “Staphylococcus aureus” (https://www.ncbi.nlm.nih.gov/protein/?term=myosin+AND+"Staphylococcus+aureus") on August 5, 2019. The rspbproc utility available at the CDD FTP site was the version released June 29, 2015. The searches were carried out in August 2019 against CDD database version 3.17, released April 3, 2019. Please note that using updated versions of the CDD database, RPS‐BLAST, and rpsbproc utility may result in slightly different results.

The CDD predicts domains on your protein(s) of interest and provides important clues about its function. To pursue options for further analysis, readers are encouraged to launch SPARCLE (the Subfamily Protein Architecture Labeling Engine; see Guidelines for Understanding Results section on Basic Protocol 1) from the domain architecture ID link, on the CD‐Search Results page (Figure 5), to investigate further protein classification. SPARCLE is a CDD resource that allows comparative analyses of protein families on the basis of conserved domain architecture and for the functional characterization and labeling of protein sequences that have been grouped by their characteristic conserved domain architecture. SPARCLE can also be accessed directly from the SPARCLE home page (https://www.ncbi.nlm.nih.gov/sparcle). For example, you could search in SPARCLE/advanced search builder with "Myosin" in the name field. Detailed SPARCLE help is available by clicking the question mark box on the SPARCLE results page.

The three CD‐Search protocols in this paper describe querying a single protein and large numbers of proteins, interacting with the CDD though its web interfaces or programmatically. You may also want to try Batch CD‐Search as an interface for scripted data retrieval. A query can be submitted as either an HTTP GET or an HTTP POST request. An HTTP GET request is submitted as a URL. The program performs the search, collects all the data into a master data structure, and extracts the subset of information you have requested for the final output. The Base URL, valid parameters, and examples of URLs for HTTP GET requests, as well as sample PERL scripts for HTTP POST operations, can be found at: https://www.ncbi.nlm.nih.gov/Structure/cdd/cdd_help.shtml#BatchRPSBWebAPI

### Time Considerations

Note that unlike running CD‐Search and Batch CD‐Search, running RPSBLAST is time consuming. It takes 2 s on average to process one protein or nucleotide sequence; thus, for instance, if you have 10,000 sequences in your FASTA file, it may take 5 to 6 hr to finish. However, the rpsbproc processing is fairly quick: it takes only >30 s to process the RPS‐BLAST output of 10,000 protein sequences.

### Troubleshooting

Help documentation is provided in Table 1.
