# Supplementary material for: NCBI's Conserved Domain Database and Tools for Protein Domain Analysis
Source: Curr Protoc Bioinformatics. 2019 Dec 18;69(1):e90. doi: 10.1002/cpbi.90 (PMC7378889; doi:10.1002/cpbi.90)
Supplement: Supplementary file 1 [file CPBI-69-0-s001.docx]

CAA37068.1

CAA32167.1

A23662

A41604

P19524.1

P12844.1

P02563.2

P02564.2

P10569.1

P12847.1

P10568.1

P05659.1

P14105.1

P24733.1

AAA28686.1

AAA28687.1

AAA28713.1

AAA28718.1

AAA28719.1

AAA28720.1

AAA28721.1

AAA29905.1

AAA33201.1

AAA51837.1

AAC17185.1

AAA37159.1

AAA37161.1

AAA48985.1

AAA48986.1

AAA48987.1

BAA00791.1

AAB19994.1

CAA79675.1

P32492.1

S33812

AAC46490.1

Q02440.1

AAA59888.1

CAA86293.1

AAA99177.1

P08964.3

AAB09051.1

AAC50402.1

P19706.2

BAA08111.1

P02565.3

P47808.1

P35748.2

AAB03660.1

AAB03661.1

AAB09048.1

AAB09050.1

AAB48065.1

CAA69352.1

2MYS_A

BAA19691.1

AAB53061.1

AAB53062.1

Q04439.1

Q02566.2

P13535.3

P13540.2

AAC40124.1

AAC24207.1

AAC27525.1

1BR1_A

1BR2_A

P10587.4

P13539.2

BAA34954.1

AAD29948.1

AAD29949.1

AAD29951.1

AAD33718.1

1B7T_A

AAD52842.1

CAB36794.2

P35579.4

AAF05903.1

AAF05904.1

BAA87057.1

AAF20150.1

AAF25688.1

AAF25689.1

NP_034986.1

1D0X_A

1D1C_A

NP_036736.1

NP_037326.1

AAF34810.1

CAB56466.2

AAF36524.1

AAF37875.1

T30148

AAF70861.1

AAF71540.1

AAC47725.3

AAC47726.3

AAF72176.1

NP_058936.1

AAF78910.1

BAB00612.1

AAF68025.2

CAC05419.1

AAG24560.1

A59234

A59249

1FMV_A

1DFK_A

1DFL_A

AAG43570.1

CAC20413.1

AAG49341.1

Q9USI6.1

NP_002464.1

1G8X_A

Q23978.1

Q9UKN7.1

NP_074035.1

NP_002465.1

AAK17202.1

BAB39452.1

P70569.1

Q05096.1

Q62774.1

Q62812.3

Q9JLT0.1

Q9QYF3.1

O08638.1

P70248.1

Q27991.2

Q28641.1

Q64331.1

Q90339.2

Q9UBC5.1

Q9UKX2.1

P47807.2

P13538.4

P35749.3

BAB40711.1

1I84_S

NP_113708.1

AAH08538.1

Q63358.1

Q9QY06.2

O14157.1

AAK92927.1

AAL30896.1

1JWY_A

P79114.1

NP_490755.1

NP_490856.1

CAC85955.1

AAL78671.1

NP_542766.1

NP_584783.1

AAL91722.1

AAH26142.1

NP_620248.1

AAM50150.1

1LKX_A

AAM77560.1

AAM77561.1

EAA20190.1

AAN36373.1

Q63356.1

1M8Q_A

NP_523503.2

NP_723271.1

NP_723999.1

NP_724000.1

NP_724001.1

NP_724003.1

NP_724004.1

NP_724005.1

NP_724006.1

NP_724007.1

NP_724008.1

NP_724009.1

NP_724010.1

NP_523860.2

NP_726506.1

1KK7_A

NP_508504.2

AAN75148.1

BAC30831.1

BAC32206.1

BAC32210.1

BAA34519.2

BAA74889.2

BAA96036.2

NP_776819.1

NP_777259.1

AAH45324.1

NP_523587.4

AAH47253.1

AAO52485.1

CAD70976.1

NP_082297.1

BAA74888.2

AAH49849.1

CAD91136.1

O94477.1

O94832.2

CAD98272.1

NP_506065.2

NP_501620.2

Q8IUG5.1

AAP88402.1

AAP88403.1

CAE11864.1

Q92614.3

AAO39147.1

NP_780469.1

AAH57729.1

AAQ87014.1

AAQ88310.1

AAQ88311.1

1OE9_A

AAH60471.1

AAH61145.1

AAH60675.1

P34109.2

AAH18933.2

1QVI_A

Q23979.3

NP_776542.1

NP_777152.1

NP_175453.2

NP_990097.1

NP_990805.1

NP_990808.1

NP_990605.1

NP_991142.1

EAK84273.1

O74805.1

EAK91894.1

Q8K3H5.1

AAT12332.1

CAF94090.1

CAF97644.1

CAG10354.1

AAH72844.1

AAH73455.1

O00934.1

O00936.4

BAD32323.1

NP_001001302.1

AAH79699.1

AAT92220.1

Q99323.2

P12845.2

CAH90560.1

CAH91418.1

NP_999020.1

NP_999301.1

Q875Q8.1

Q875X3.2

Q875X4.2

Q876G9.2

CAH93643.1

NP_001009221.1

Q9Y7Z8.1

Q7RQ71.1

Q8IDR3.1

Q9XYF6.1

EAL63071.1

EAL69262.1

AAH90979.1

1W8J_A

NP_055826.1

NP_001014552.1

NP_001014553.1

1YV3_A

NP_001019619.1

XP_636580.1

XP_643200.1

AAY63881.1

NP_034985.2

XP_678702.1

AAY86556.1

BAE06108.1

O43795.3

BAE07187.1

2BKI_A

BAE16257.1

BAE16258.1

BAE16259.1

BAE16270.1

NP_796343.2

Q6URW6.1

Q61879.2

NP_492186.3

NP_001022590.1

NP_001023548.1

2BKH_A

P13542.2

P10676.2

Q5SX40.1

Q5SX39.1

BAE34176.1

BAE27768.1

BAE27998.1

BAE24986.1

BAE23716.1

ABA01554.1

Q7Z8J6.1

Q6C7C0.1

Q6CVE9.1

Q6FMJ3.1

Q6FN18.1

Q59MQ0.1

Q7SDM3.1

Q4P9K9.1

Q622K8.1

Q60LV4.1

Q9TW28.1

Q9XXV8.1

Q86AC8.1

Q9V3Z6.1

P91443.1

Q9U1M8.1

Q29122.1

Q8MJU9.1

Q8MJV0.1

Q8MJV1.1

Q9BE39.1

Q9BE40.2

Q9BE41.1

Q9TV61.1

Q9TV62.1

Q9TV63.1

Q9LKB9.1

Q39160.1

Q9M2K0.1

Q9LHE9.1

Q5ZMC2.1

2AKA_A

ABB70410.1

Q5SV80.1

Q9ERC1.1

Q91Z83.1

Q5SUA5.1

Q5SYD0.1

Q9Z1N3.1

Q5ZLA6.1

Q92002.1

Q9I8D1.1

Q569U0.1

NP_109604.1

XP_728625.1

P12883.5

AAI10701.1

ABC40752.1

ABC40753.1

ABC40754.1

Q63357.3

AAK97502.3

NP_001035202.1

NP_001035203.1

1W9I_A

1W9J_A

1W9K_A

1W9L_A

CAK10917.1

NP_002461.2

2DFS_A

NP_062198.1

EAT35379.1

EAT36479.1

EAT38083.1

EAT39611.1

EAT42758.1

EAT44232.1

EAT48353.1

AAI14546.1

AAI17691.1

Q29RW1.1

NP_060003.2

NP_003793.2

P05661.4

NP_071855.2

NP_000248.2

NP_005954.3

NP_001070262.1

NP_001070263.1

ABJ53201.1

ABJ53202.1

NP_079005.3

NP_001070654.1

NP_057323.3

EAW14192.1

EAW53927.1

EAW53928.1

EAW55663.1

EAW55665.1

EAW55666.1

EAW66151.1

EAW66155.1

EAW71838.1

EAW71839.1

EAW71841.1

EAW71843.1

EAW76236.1

EAW76238.1

EAW90000.1

EAW90004.1

EAW90006.1

EAW90008.1

EAW90009.1

EAW90050.1

EAX08017.1

EAX08018.1

AAI27416.1

AAI29314.1

Q0CEX5.1

Q2HDI2.1

Q2US45.1

Q1DLP2.1

P13541.2

P21271.2

Q9UM54.4

Q17LW0.1

Q29P71.1

Q076A3.1

Q076A4.1

Q076A5.1

Q076A7.1

Q258K2.1

Q17R14.1

Q0WPU1.1

NP_001074719.1

NP_001074866.1

Q076A6.2

Q9JMH9.2

P49824.3

P79293.2

NP_001075227.1

NP_001075228.1

NP_001075229.1

NP_001075777.1

NP_062345.2

P08799.3

CAM71150.1

ABO96565.1

XP_001442132.1

XP_001457718.1

NP_001078847.1

ABQ08059.1

NP_001086419.1

NP_001089346.1

NP_001084034.1

ABQ59035.1

ABQ96867.1

EDK97391.1

EDL01222.1

EDL01223.1

EDL06136.1

EDL10431.1

EDL10432.1

EDL10453.1

EDL22740.1

EDL22741.1

EDL36311.1

EDL36314.1

Q5DU14.2

AAI46792.1

EDL84098.1

EDL85905.1

EDM04652.1

EDM04782.1

EDM04816.1

EDM07465.1

EDM14211.1

ABR21557.1

2OS8_A

NP_055796.1

AAI50286.1

O88329.2

Q9Y6X6.3

BAF73720.1

AAI51243.1

NP_001093582.1

NP_001093105.1

NP_002463.2

NP_036466.2

NP_002462.2

EDO16376.1

NP_001095305.1

NP_001095597.1

EDO41819.1

EDO43631.1

EDO45563.1

NP_034992.2

NP_874357.2

NP_001096641.1

EAA27097.2

NP_001098421.1

1LVK_A

1MMA_A

1MMD_A

1MMG_A

1MMN_A

NP_001103286.1

BAF84298.1

EDP36007.1

Q8NEV4.2

NP_001104237.1

2V26_A

Q9QZZ4.2

NP_001094160.1

2VAS_A

EDQ57290.1

EDQ87053.1

EDQ88212.1

EDQ88665.1

EDQ89817.1

EDQ89973.1

EDQ90868.1

EDQ90934.1

EDQ91224.1

EDQ91661.1

EDQ92819.1

NP_001107184.1

NP_001107183.1

NP_001107189.1

P54697.2

Q03479.2

P54695.2

P42522.2

P22467.2

P34092.2

BAA76844.3

XP_001743779.1

XP_001746075.1

XP_001747308.1

XP_001748290.1

EDS27056.1

EDS44957.1

2EC6_A

AAI60747.1

ACB05996.1

CAP70250.1

NP_001116613.1

Q6PIF6.2

BAG30740.1

AAI66177.1

NP_058935.2

AAI66736.1

AAI50740.1

AAI50738.1

AAI58071.1

EDU47911.1

CAO78752.1

ACD68201.1

ACD68202.1

Q8N1T3.2

Q96H55.2

Q9D6A1.2

NP_001070703.1

AAI62152.1

AAI63575.1

EDK39313.2

A2R5J1.1

B0CRJ3.2

Q00647.2

A8N2Y6.2

A6SED8.2

B0Y9Q4.2

A1C4A5.2

A5E4A8.1

A4RE77.1

A7EK16.1

A7TDZ8.1

A6ZZJ1.1

A1DBH2.1

A3LYL7.2

A8PWF6.2

EDV22061.1

EDV24561.1

EDV25275.1

EDV27005.1

EDV27755.1

EDV28363.1

EDV31450.1

NP_001122117.1

EDW09282.1

EDW12656.1

EDW61817.1

CAQ40938.1

BAG64451.1

BAG64980.1

BAG65583.1

BAG61415.1

AAI68016.1

XP_002122715.1

ACH92815.1

Q8VDD5.4

Q9HD67.3

A2AQP0.1

Q8C170.2

P02567.3

NP_001034634.2

NP_001128629.1

NP_001128630.1

3DTP_B

ACI65950.1

XP_002142647.1

EEA28731.1

CAP80800.1

NP_001101264.2

EEB07343.1

EEB10549.1

EEB11219.1

EEB12738.1

EEB12784.1

EEB15037.1

EEB16634.1

EEB16856.1

EEB20247.1

AAI70424.1

Q12965.2

P35580.3

EEC11821.1

EEC17812.1

EEC42595.1

EEC43809.1

EEC47670.1

Q6BUQ2.2

Q1EG27.2

ACL68762.1

2JHR_A

EED86499.1

EED86775.1

EED87074.1

EED88267.1

EED88551.1

EED88779.1

EED94390.1

EED94486.1

EED95019.1

EED95808.1

XP_002261533.1

NP_572669.2

NP_001036269.2

EEE20315.1

EEE23373.1

EEE28430.1

EEE30682.1

ACM48351.1

A5DKH0.3

XP_002192259.1

Q9Y623.2

NP_001139281.1

EEH53664.1

EEH57614.1

ACO63127.1

Q9WTI7.2

P12882.3

Q27966.3

A0MP03.1

A5PF48.1

Q63355.2

EEN47939.1

EEN49122.1

EEN68574.1

EEN68654.1

EEN69078.1

EEQ40783.1

ACS35536.1

ACS35537.1

ACS35540.1

ACS35543.1

ACS35545.1

NP_038635.2

NP_001155247.1

P11055.3

NP_001156538.1

AAN35999.2

3I5G_A

NP_001157207.1

NP_079690.2

NP_001157208.1

NP_001019672.2

EEU34371.1

CAX83002.1

BAI44326.1

NP_001159682.1

NP_001159699.1

ACX54880.1

CBH18711.1

EEY53382.1

EEY54365.1

EEY55432.1

EEY57494.1

EEY57772.1

EEY57949.1

EEY58105.1

EEY59580.1

EEY59915.1

EEY60594.1

EEY61076.1

EEY61222.1

EEY61268.1

EEY61334.1

EEY64252.1

EEY65764.1

EEY66782.1

EEY67460.1

EEY69399.1

EEY69764.1

EEY69816.1

O00160.3

EEZ98246.1

EEZ98803.1

EFA01220.1

EFA03648.1

EFA06086.1

EFA06975.1

EFA07932.1

EFA08290.1

EFA08775.1

EFA75546.1

EFA81458.1

EFB16037.1

EFB25464.1

EFB26994.1

NP_001162990.1

NP_001162992.1

NP_001093955.1

NP_001164071.1

BAI63631.1

BAI63632.1

BAI63633.1

BAI63634.1

BAI63635.1

EFC39236.1

EFC48085.1

EFC48823.1

XP_002680898.1

ADD91458.1

2W4A_M

Q9NQX4.2

ADG63228.1

Q9UKN7.2

Q9Y4I1.2

B2RTY4.2

Q9ULV0.3

Q8WXR4.4

Q9Y2K3.5

DAA15521.1

DAA18731.1

DAA18765.1

DAA18801.1

DAA19711.1

DAA25710.1

DAA29476.1

DAA33524.1

3MYH_X

CBJ28361.1

CBJ30507.1

CBJ31129.1

CBJ31893.1

CBJ27150.1

CBJ27565.1

CBJ27624.1

CBN74726.1

CBN73968.1

CBN73971.1

CBN79332.1

CBN77168.1

CBK23300.2

EFJ05534.1

EFJ20281.1

NP_001179691.1

XP_002900773.1

XP_002907688.1

CBI20376.3

NP_983470.2

ADM15669.1

NP_001182221.1

NP_001182222.1

EFN58364.1

EFN63268.1

EFN63513.1

EFN72913.1

EFN74075.1

EFN74640.1

EFN75980.1

EFN81262.1

EFN84300.1

EFN85624.1

EFN88457.1

EFN88873.1

NP_001182585.1

P54696.3

EFO89684.1

CAP35022.2

NP_001185879.1

EFR23843.1

CBY30341.1

CBY07869.1

CBY09893.1

CBY11189.1

CBY11434.1

3L9I_A

ADU19853.1

B0I1T2.2

A7E2Y1.3

O00159.4

P13533.5

CBN81020.1

NP_001091647.2

2X51_A

EFW39854.1

EFW40315.1

EFW41160.1

EFW42657.1

EFW43530.1

EFW45955.1

EFW47268.1

NP_001189009.1

NP_001189011.1

NP_001188729.1

NP_001188730.1

EFX66210.1

EFX67125.1

EFX69901.1

EFX72449.1

EFX76460.1

EFX77076.1

EFX78767.1

EFX79888.1

EFX87104.1

EFX89508.1

Q9UKX3.2

EFZ11166.1

EFZ14256.1

EGB03856.1

EGB06001.1

EGB06809.1

EGB06912.1

EGB08826.1

EGB09584.1

EGB10675.1

EGB11060.1

EGB11823.1

EGB12388.1

EGB13090.1

EGB13170.1

ADY39846.1

ADY39973.1

ADY39976.1

ADY39989.1

ADY40009.1

ADY40035.1

ADY40738.1

ADY41485.1

EGC28970.1

EGC34012.1

EGC40078.1

CCA14100.1

CCA15095.1

CCA15693.1

CCA16029.1

CCA16530.1

CCA16732.1

CCA16800.1

CCA17136.1

CCA18292.1

CCA18606.1

CCA19359.1

CCA20290.1

CCA21290.1

CCA23203.1

CCA24180.1

CCA26006.1

CCA25743.1

Q13459.3

EGD73038.1

EGD74581.1

EGD75567.1

EGD75829.1

EGD76052.1

EGD76304.1

EGD77017.1

EGD77364.1

EGD77790.1

EGD78782.1

EGD78899.1

EGD79108.1

EGD79713.1

EGD80527.1

EGD81162.1

EGD81471.1

EGD81714.1

EGD82520.1

EGD83184.1

XP_003217132.1

XP_003217135.1

Q7Z406.2

EGF77626.1

EGF79520.1

EGF82537.1

EGG07707.1

EGG14947.1

EGG24917.1

NP_001193103.1

3MNQ_A

EGI58350.1

EGI59169.1

EGI59287.1

EGI60548.1

EGI69530.1

2XO8_A

EGN94962.1

EGO01283.1

EGO25918.1

P0CP01.1

XP_003372801.1

CCC54238.1

XP_003388016.1

EGR34364.1

2XEL_A

2Y0R_X

2Y9E_X

EGT30557.1

EGT39176.1

Q99104.2

P97479.2

Q99MZ6.3

P46735.3

EGU12695.1

EGV92993.1

EGV94928.1

EGW01271.1

EGW06670.1

EGW15128.1

AEO67085.1

P36006.4

XP_003442528.1

XP_003443121.1

XP_003447953.1

XP_003451049.1

EGZ05166.1

EGZ09686.1

EGZ11476.1

EGZ15515.1

EGZ15946.1

EGZ16878.1

EGZ20778.1

EGZ25771.1

EGZ26409.1

EGZ28124.1

EGZ29818.1

EHA97370.1

EHB02939.1

EHB04540.1

EHB04541.1

EHB04543.1

EHB04546.1

EHB06428.1

EHB10634.1

EHB17149.1

CCA70724.1

CCA72950.1

CCD42040.1

CCD42041.1

CCD42042.1

Q01989.4

EHH20189.1

EHH24515.1

EHH24530.1

EHH24531.1

EHH24533.1

EHH27757.1

EHH27758.1

EHH54168.1

EHH57731.1

EHH57733.1

EHH58705.1

3J04_A

CCE63324.1

EHJ64330.1

EHJ66585.1

EHJ67502.1

EHJ67857.1

EHJ74610.1

EHJ77507.1

EHJ78183.1

EHJ78713.1

EHJ78837.1

EHJ79042.1

GAA96153.1

GAA96264.1

GAA36277.2

CCD82429.1

XP_416950.3

XP_001231456.2

XP_427876.3

NP_001242941.1

NP_005955.3

NP_001243024.1

4DB1_A

E9Q634.1

EHY65485.1

AFE63991.1

AFE65943.1

F8VQB6.1

D3ZJP6.1

AFH27291.1

AFH34098.1

XP_003707687.1

EIE22795.1

EIE25498.1

EIE90774.1

EIE92316.1

AFI33494.1

AFI37202.1

AFI37204.1

AFI38373.1

EIF49478.1

NP_001246516.1

NP_724002.2

NP_001246048.1

NP_001246049.1

NP_001246050.1

NP_001246051.1

CCH58825.1

EIM20765.1

EIM20792.1

EIM22427.1

EIM24233.1

GAB67643.1

GAB67768.1

EIM88048.1

EIN11094.1

XP_003739851.1

EIW68685.1

EIW68985.1

EIW76465.1

EIW83186.1

NP_001257157.1

EJD01749.1

EJD36764.1

EJD51949.1

EFO23663.2

EJD76471.1

EJD76606.1

AFN83815.1

EJK64551.1

EJK69525.1

4A7F_C

CCE31454.1

CCF73982.1

CCF74513.1

CCF74947.1

EJT49432.1

EJU01525.1

EJU06309.1

AFQ62077.1

EJW85906.1

CCK70230.1

CCK70935.1

BAM38975.1

BAM39265.1

EJY72420.1

AFR96137.1

4E7S_A

4E7Z_A

EKC19757.1

EKC20877.1

EKC28928.1

EKC30215.1

EKC30269.1

EKC32141.1

EKC33506.1

EKC35139.1

EKC37566.1

EKC39652.1

EKC41639.1

CCH46395.1

AFS69160.1

EKF27391.1

EKF32066.1

EKF32117.1

EKF32619.1

EKF32816.1

EKF38939.1

EKF39180.1

EKG03591.1

EKG14177.1

AFU81219.1

NP_001258467.1

NP_001258469.1

EKM60048.1

JAA42963.1

JAA42964.1

JAA42965.1

JAA42967.1

JAA42969.1

JAA42970.1

XP_003964373.1

XP_003965022.1

XP_003972149.1

XP_003972534.1

XP_003973714.1

XP_003977677.1

XP_003978631.1

CCO17325.1

CCO16900.1

JAA50057.1

JAA50060.1

JAA50065.1

JAA53320.1

JAA53322.1

JAA53323.1

JAA53670.1

JAA59614.1

JAA59623.1

JAA61409.1

JAA63808.1

EKX34711.1

EKX73815.1

EKX74322.1

AFZ80026.1

ELA47849.1

CCJ30164.1

ELK03857.1

ELK03872.1

ELK03874.1

ELK03877.1

ELK10272.1

ELK11239.1

ELK11626.1

ELK24949.1

ELK29644.1

ELK31839.1

ELK31856.1

ELK31861.1

ELK33290.1

ELK35035.1

XP_004065556.1

XP_004066249.1

XP_004071152.1

XP_004071883.1

XP_004078214.1

ELP90006.1

ELR10801.1

ELR14460.1

ELR21237.1

ELR21917.1

ELR25680.1

ELR47752.1

ELR48000.1

ELR48002.1

ELR48945.1

ELR50107.1

ELR54390.1

ELR58852.1

Q758Q9.2

ELT88116.1

ELT99384.1

ELT99775.1

ELU38612.1

4DBP_A

4DBQ_A

4DBR_A

ELW47118.1

ELW53081.1

ELW53084.1

ELW53086.1

ELW64891.1

ELW67681.1

EMC87130.1

EMC88972.1

EMC96859.1

AGE96299.1

EMD36962.1

EMD40311.1

EME86661.1

EMF14900.1

Q13402.2

CCU97669.1

EMP28603.1

EMP33063.1

XP_004349077.1

EMR86891.1

EMS22032.1

EMS26028.1

ENH88047.1

ENN70532.1

ENN71157.1

AGI96978.1

EOB05137.1

EOB07817.1

EOD45895.1

EON63727.1

XP_004571949.1

XP_004571951.1

CCG84382.1

CCG81796.1

Q8SS35.2

EPB84924.1

EPB92762.1

XP_002933065.2

XP_002937051.2

EFW47369.2

EFW45016.2

EPH53635.1

F4IVR7.1

F4HWY6.1

F4IRU3.1

F4IUG9.1

F4JM19.1

F4K0A6.1

F4I507.1

F4JIU4.1

F4I5Q6.1

F4I460.1

F4HXP9.1

F4K5J1.2

EPQ25921.1

EPQ25956.1

EPQ50784.1

EPQ54333.1

EPQ58854.1

CCM26457.1

EPR58865.1

EPR61337.1

EPR63013.1

EPR64107.1

EPR79697.1

XP_005104354.1

EPS70501.1

EPS70842.1

EPT00859.1

EPX72489.1

EPY22327.1

EPY29051.1

XP_005163758.1

XP_001920958.3

XP_005172645.1

EPY89280.1

EQC25464.1

EQC25796.1

EQC26484.1

EQC28326.1

EQC28329.1

EQC28330.1

EQC29751.1

EQC29888.1

EQC30122.1

EQC31381.1

EQC35694.1

EQC35749.1

EQC35852.1

EQC35904.1

EQC36197.1

EQC36789.1

EQC37560.1

EQC38890.1

EQC39065.1

EQC39241.1

EEB07886.2

ERE76313.1

ERE76407.1

ERE91016.1

BAN82211.1

BAN82054.1

ERF72609.1

XP_005585015.1

XP_005585016.1

ERL86506.1

XP_005746451.1

XP_005693420.1

ERT01548.1

XP_005797606.1

XP_005802858.1

XP_005805036.1

XP_005821965.1

XP_005826513.1

XP_005838009.1

ESA01981.1

EAA35476.3

XP_005915364.1

XP_005924208.1

ESK91869.1

NP_001273098.1

ESN91883.1

ESN99871.1

ESO04701.1

ESO06156.1

CDJ12647.1

ESO82004.1

ESO82465.1

ESO85384.1

ESO87527.1

ESO87529.1

ESP01302.1

ESP05442.1

CDJ19304.1

XP_005986736.1

XP_006001756.1

CDI77167.1

CDJ37806.1

CDJ39225.1

CDJ56122.1

CDJ31293.1

CDJ58648.1

CDJ60330.1

CDJ97549.1

CDJ95274.1

CDJ89635.1

ESW05138.1

XP_006258441.1

ETB56952.1

ETB61800.1

ETB62830.1

ETB63451.1

ETI49158.1

CDG41623.1

ETK77196.1

ETL30030.1

ETL83261.1

ETL95628.1

ETM48823.1

ETN18268.1

ETN46065.1

ETN65570.1

ETN76983.1

XP_006542969.1

XP_006542971.1

XP_006534683.1

XP_006534685.1

ETO77893.1

ETO85300.1

ETP31910.1

ETP46875.1

AHF46691.1

XP_006639635.1

ETW03251.1

ETW31661.1

4L79_A

EUT90152.1

XP_006784759.1

XP_006825854.1

P02566.2

XP_006940689.1

XP_007091372.1

4BYF_A

NP_065935.3

XP_007432200.1

XP_007490131.1

XP_007641525.1

XP_007895376.1

XP_008179213.1

XP_008179221.1

4QBD_A

KEG00372.1

KEG02844.1

XP_008282168.1

XP_008301730.1

XP_008436352.1

XP_008554215.1

XP_008626742.1

BAL60532.2

XP_008895963.1

CDR10555.1

CDR13691.1

CDS44324.1

CDS48035.1

CDU15967.1

CDU18738.1

CDZ10488.1

XP_008947752.1

XP_008964179.1

KFO15496.1

KFP88850.1

XP_009303854.1

XP_009558586.1

XP_009835964.1

XP_009968214.1

XP_010754160.1
